# Comparative Analysis of Transcription Factor Binding Sites in the Long Control Region Across Human Papillomavirus Types

**DOI:** 10.3390/v18060646

**Published:** 2026-06-04

**Authors:** Derrin Bright, Juan I. Fuxman Bass

**Affiliations:** 1 Vellore Institute of Technology, School of Bio Sciences and Technology, Vellore 632014, Tamil Nadu, India; derrin.bright2022@vitstudent.ac.in; 2 Biology Department, Boston University, Boston, MA 02215, USA; 3 Bioinformatics Program, Boston University, Boston, MA 02215, USA; 4 Molecular Biology, Cell Biology and Biochemistry Program, Boston University, Boston, MA 02215, USA

**Keywords:** papillomavirus, long control region, transcription factor, HPV

## Abstract

Human papillomaviruses (HPVs) comprise more than 200 types associated with diverse clinical outcomes, ranging from benign lesions caused by low-risk types to cancers driven by high-risk types. These differences are partly driven by variation in the Long Control Region (LCR), a non-coding element that regulates viral gene expression through interactions with viral and host transcription factors (TFs). Although individual TF binding sites have been mapped in a few well-studied HPV types, the broader regulatory differences between high-risk and low-risk HPVs remain poorly defined. Here, we systematically analyzed LCR sequences from 207 HPV types using TF motif scanning and identified 104 TFs with significantly different binding site densities between risk groups. Integration with TCGA transcriptomics data showed that 50 of 69 TF enriched in high-risk types are expressed in HPV-positive head and neck tumors (HNSC) and 53 in HPV-positive cervical tumors (CESC). Analysis of published ChIP-seq datasets further confirmed LCR occupancy for seven of these TFs in HPV18-positive cells. In addition, conservation analysis across clinical isolates of HPV-16 and HPV-18 identified highly conserved TF binding sites overlapping multiple high-risk-enriched TF motifs, suggesting functional constraint on key regulatory elements. Together, these findings reveal distinct TF binding landscapes associated with HPV risk groups and identify candidate host regulators that may contribute to differences in viral transcriptional programs and oncogenic potential across HPV types.

## 1. Introduction

Human papillomaviruses (HPV) are one of the most prevalent infectious threats to human health globally. Persistent infection with high-risk HPV genotypes is the necessary cause of virtually all cervical cancers, with 99.7% of invasive cases testing positive for HPV DNA, and is highly prevalent in multiple other cancers [1,2]. Over 200 HPV types have been characterized, with differing oncogenic potential and tissue tropism [3]. Low-risk HPV types cause benign lesions, most commonly genital warts or papillomatosis, whereas high-risk types are oncogenic and drive cervical as well as a substantial fraction of oropharyngeal, vulvar, vaginal, penile, and anal cancers [4].

The HPV genome is a double-stranded circular DNA of approximately 8 kb, comprising early genes (E1, E2, E4, E5, E6, E7) and late capsid proteins (L1, L2) under the regulatory control of a non-coding element commonly called the Long Control Region (LCR) [5]. The LCR is located between the L1 and E6 open reading frames and spans approximately 400–1000 bp, depending on the HPV type. This region contains the viral origin of replication, the early promoter containing a set of transcription factor (TF) binding sites that control when and where viral genes are expressed. The LCR drives expression of E6 and E7, whose constitutive activity is necessary and sufficient for maintaining the transformed phenotype in HPV-positive cancers [6]. LCR-driven transcription, however, operates differently across HPV types. In low-risk types such as HPV-11, the LCR is tightly coupled to epithelial differentiation, with activity suppressed in basal cells and induced only in the differentiating spinous layers [7]. Viral integration in high-risk types disrupts the E2 open reading frame, removing its repressive function on the LCR and resulting in overexpression of E6/E7 [8]. The LCR also encodes tissue tropism through its enhancer composition, with comparative studies across 14 HPV types showing that genital types, both high- and low-risk, share strong epithelial-specific enhancers, whereas cutaneous types show lower LCR activity [9,10].

Biochemical and reporter assay studies have identified several host TFs that bind and regulate LCR activity. The AP-1 family (Jun/Fos heterodimers) was among the first characterized, with the HPV-16 LCR containing three AP-1 binding sites that regulate early transcription, together with SP1 [11,12]. Conversely, YY1 and POU2F1 suppress LCR transcription [13,14]. CEBPB, instead, activates or represses the HPV-16 LCR depending on which isoform is expressed [15]. Together, these studies have provided valuable insight into the mechanisms of LCR regulation; however, most are focused on a single or a few HPV types, mainly HPV-16 and HPV-18. As a result, the full range of TFs that regulate LCR activity across the papillomavirus family remains unknown.

A key unresolved question is whether TF binding patterns in the LCR differ systematically between high-risk and low-risk HPV types, and, if so, which regulators underlie these differences. Such differences could reflect adaptation to distinct epithelial niches, differentiation states, persistence strategies, or host transcriptional environments, while also contributing indirectly to differences in oncogenic potential. Although prior studies have documented sequence variation across HPV LCRs and characterized selected TF binding sites in a limited number of genotypes [16], a comprehensive, LCR-wide analysis of TF binding enrichment across the full diversity of classified HPV types is lacking. Moreover, the extent to which predicted TF binding sites are conserved across clinical isolates within the same HPV type, an expectation for functionally important elements, has not been systematically evaluated.

Here, we compared TF binding sites across the LCRs of 207 HPV types and found that high-risk and low-risk types recruit substantially different sets of host TFs. Of the 104 differentially enriched TFs, 69 showed greater predicted binding density in high-risk LCRs and 35 in low-risk LCRs. A total of 50 of the 69 high-risk TFs were expressed in HPV-positive head and neck squamous cell carcinoma (HNSC) tumors and 53 in HPV-positive cervical cancer (CESC) tumors from the Cancer Genome Atlas (TCGA), suggesting they have the potential to regulate viral transcription in the context of HPV-associated cancer. The remaining TFs include nine composite heterodimer motifs and factors with low or tissue-restricted expression. Seven high-risk TFs were independently confirmed to physically occupy the HPV-18 LCR by ChIP-seq in HPV-18-positive cells. Eight TFs were conserved across ≥90% of both HPV-16 and HPV-18 clinical isolates, pointing to a shared core regulatory program across the two most clinically relevant high-risk types. Finally, we also identified multiple TF sites differentially enriched in the late promoter between high- and low-risk HPV types. Together, these results suggest potential host factors linked to transcriptional differences between HPV types.

## 2. Materials and Methods

### 2.1. HPV Genome Collection and LCR Extraction

Complete HPV genome sequences for 207 HPV types were obtained from the Papillomavirus Episteme (PaVE) database and NCBI GenBank (Table S1). Risk classification (27 high-risk; 180 low-risk) was assigned according to the International Agency for Research on Cancer (IARC) Monographs and literature survey. LCR sequences were extracted computationally by identifying the genomic interval between the stop codon of the L1 open reading frame and the start codon of the E6 open reading frame. LCR lengths ranged from 322 to 979 bp (median ≈ 527 bp). For intra-type conservation analysis, 990 complete HPV-16 genomes and 187 complete HPV-18 genomes were downloaded from NCBI GenBank (Table S2). LCR extraction was performed using the same approach, resulting in 835 HPV-16 LCRs (155 excluded due to incomplete L1/E6 annotations) and 187 HPV-18 LCRs.

### 2.2. Transcription Factor Binding Site Prediction

TF binding sites were identified using the Find Individual Motif Occurrences (FIMO) tool from the MEME Suite [17]. Scanning was performed using all 892 human TF position weight matrices from the JASPAR database [18] with the following parameters: *p*-value threshold of 1 × 10^−4^, both DNA strands scanned, and 0th-order Markov background model computed from the nucleotide frequencies of all input LCR sequences. To account for the substantial variation in LCR length across HPV types, binding site counts were normalized to binding site density (sites per kilobase), calculated as:

Density = (number of binding sites)/(LCR length in kb)

This prevents length-dependent bias in which longer LCRs would accumulate more binding sites irrespective of true biological enrichment. To further explore enrichment patterns within a single genus, the analysis was also performed using only alpha-papillomavirus LCR sequences, comparing high-risk and low-risk alpha types directly. Of the 27 types classified as high risk, all but one (HPV-5) belong to alpha-papillomavirus. The motif analysis is provided in Table S3.

### 2.3. Statistical Analysis of Differential TF Binding

Of the 892 TFs scanned, 756 produced at least one binding site hit across all HPV LCRs and were retained for downstream analysis; the remaining 136 TFs had zero hits. For each of these 756 TFs, binding site densities were compared between high-risk (*n* = 27) and low-risk (*n* = 180) HPV types using the two-sided Mann–Whitney U test. *p*-values were corrected for multiple testing using the Benjamini–Hochberg procedure to control the false discovery rate (FDR) at 5%. TFs with FDR-adjusted *p*-values < 0.05 were considered differentially enriched (Table S3). The mean density difference between high-risk and low-risk groups was computed for each significant TF to determine the direction of enrichment.

### 2.4. ChIP-Seq Analysis

ChIP-seq datasets for YY1, DNTTIP1, CEBPB, ELK1, ELK4, MYBL2, and MYC in HPV-18-infected HeLaS3 and HeLa cells were retrieved from ENCODE (ENCSR000EDA: CEBPB, ENCSR000ECI: ELK1, ENCSR000EVI: ELK4, ENCSR000EZD: MYC) and GEO (GSM779433: YY1, GSE235845: DNTTIP1 and GSE27030: MYBL2). Reads were aligned to the HPV-18 reference genome using Bowtie2 [19], and read coverage was calculated using SAMtools [20], normalized to reads per million mapped reads (RPM), and fold enrichment over matched input controls was computed at each position. TF occupancy within the HPV-18 LCR was assessed for all seven TFs. MYCN was enriched in high-risk HPV types but did not have available ChIP-seq data in HeLaS3 cells. However, its close paralog MYC, which contains a near-identical motif and is highly expressed in HeLa cells, was found to bind to the HPV-18 LCR.

### 2.5. TCGA-HNSC and TCGA-CESC RNA-Seq Validation

RNA-seq data from the TCGA-HNSC and TCGA-CESC projects were used for expression validation. Raw count data for 566 samples were downloaded via TCGAbiolinks [21]. After restricting to primary tumor samples with known HPV status (annotated via cBioPortal subtype classifications), 487 independent samples remained (72 HPV-positive, 415 HPV-negative) and 175 for CESC (167 HPV-positive, 8 HPV-negative). Differential expression analysis was performed using DESeq2 [22]. Genes with fewer than 10 reads in fewer than 10% of the cohort were excluded, retaining 24,282 genes for analysis. TFs were considered validated if they met two criteria: |log_2_FoldChange| > 1 and adjusted *p*-value < 0.05. Transcripts per million (TPM) values were calculated to assess expression levels. Gene lengths were obtained from Ensembl via the biomaRt package. TFs with mean TPM ≥ 1 across at least 10 percent of HPV+ patients were considered meaningfully expressed.

### 2.6. IPA Upstream Regulator Analysis

Differentially expressed genes from the TCGA-HNSC DESeq2 analysis were submitted to Ingenuity Pathway Analysis (IPA) [23] for upstream regulator prediction. TFs identified in our JASPAR analysis were cross-referenced with IPA upstream regulator results to determine whether they were independently recognized as transcriptional regulators in the HNSC dataset and to obtain predicted activation states and z-scores.

### 2.7. Intra-Type Conservation Analysis

To assess evolutionary conservation of TF binding sites, FIMO scanning was performed on 835 HPV-16 and 187 HPV-18 isolate LCRs using specific background nucleotide frequencies. Conservation percentage for each TF was defined as

Conservation (%) = (number of isolates with ≥1 binding site)/(total isolates) × 100.

A total of 30 TFs were selected for conservation analysis, comprising the top 20 high-risk-enriched TFs and the top 10 low-risk-enriched TFs, both ranked by absolute mean density difference (|Δ|), all with FDR < 0.05 in the primary JASPAR analysis (Table S3).

### 2.8. Late Promoter Analysis

We applied the same FIMO-based pipeline to the late promoter region of 220 HPV types. The HPV late promoter lies within the E7 open reading frame and is activated during the differentiation-dependent phase of the viral life cycle [3]. Late promoter sequences were defined as the full E7 ORF (261 to 348 bp, mean 295 bp) and analyzed using a sequence-specific background model. Density normalization, Mann–Whitney U testing, and Benjamini–Hochberg correction were applied as described for the LCR analysis (Table S3).

## 3. Results

### 3.1. Differential TF Enrichment Across High- and Low-Risk HPV LCRs

To determine whether the HPV LCR has different TF binding patterns between high-risk and low-risk viruses, we performed FIMO scanning of 207 HPV LCR sequences (27 high-risk, 180 low-risk) against all 892 human TFs from the JASPAR database. Of these, 756 TFs had at least one predicted binding site in at least one of the LCRs evaluated. Because HPV LCR lengths vary substantially across different types (range: 322–979 bp), raw binding site counts were normalized by LCR length. Several TFs known to regulate HPV transcription, including SP1, AP-1 and POU2F1, were detected across LCRs of both risk groups without significant differential enrichment, consistent with their shared regulatory roles in high- and low-risk HPV types [7,24,25].

**Figure 1:**
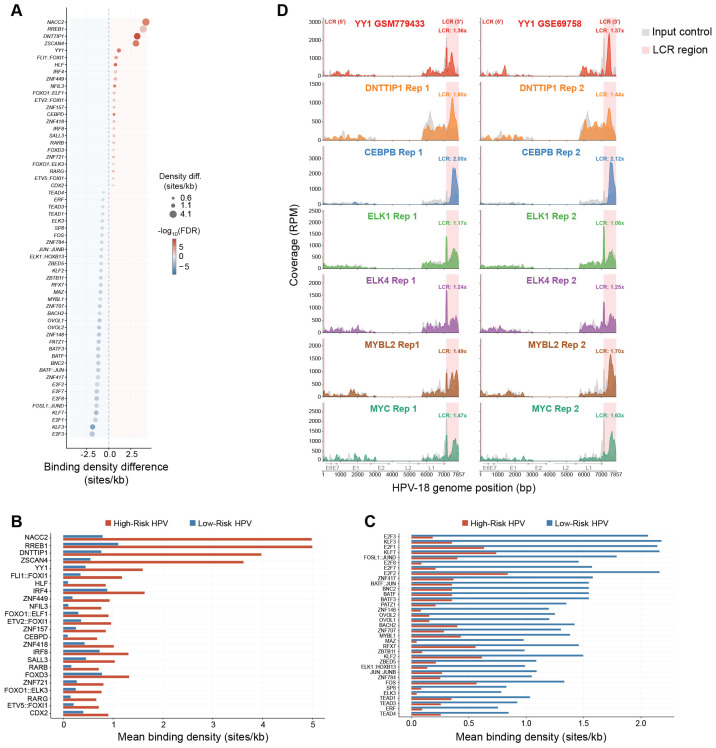
TF binding site enrichment across 207 HPV LCRs. (**A**) Dot plot showing the mean binding site density difference (Δ sites/kb; HR mean density minus LR mean density) for all TFs with |Δ| > 0.5 and FDR < 0.05. Each dot represents one TF; dot color indicates enrichment direction (red = enriched in high-risk, blue = enriched in low risk), and dot size is proportional to |Δ|. Positive values indicate higher binding density in high-risk LCRs; negative values indicate higher density in low-risk LCRs. (**B**,**C**) Bar charts showing mean predicted binding site density (sites/kb) in high-risk (red) and low-risk (blue) HPV LCRs for TFs enriched in high-risk types (**B**) or low-risk types (**C**). Only TFs with |Δ| > 0.5 sites/kb are shown. (**D**) ChIP-seq read coverage tracks across the HPV-18 reference genome for seven TFs enriched in high-risk HPV types.

Differential enrichment analysis identified 104 TFs with significantly different binding-site densities between high- and low-risk HPV LCRs, including 69 enriched in high-risk types and 35 in low-risk types (Figure 1A). The top high-risk-enriched TFs include NACC2, RREB1, DNTTIP1 and ZSCAN4 (Figure 1B). Additional high-risk-enriched TFs with notable effect sizes included YY1, a known direct binder of the HPV-18 [13,26], whose interaction with TFAP2 family members at HPV regulatory motifs was recently characterized [27]. Among low-risk-enriched TFs, the E2F and KLF families of proteins dominated the top rankings (Figure 1C).

To confirm TF binding to LCRs of high-risk types, we leveraged ChIP-seq datasets from the HPV-18-infected HeLaS3 and HeLa cells, available for 6 of the 69 high-risk TFs identified (YY1, DNTTIP1, CEBPB, ELK1, ELK4, MYBL2). All six TFs showed enrichment over matched input controls within the LCR, resulting in a 100% validation rate, with C/EBPβ showing the most pronounced LCR-specific peaks (Figure 1D). Additionally, MYC, a paralog of MYCN enriched in high-risk HPVs that shares the same binding motif, is also recruited to the HPV-18 LCR in HeLaS3 cells. These data provide experimental validation for the FIMO-derived enrichment signals and confirm that HR-enriched TF binding motifs in HPV-18 LCRs correspond to sites of physical TF occupancy in HPV-18-positive cells.

Since the high/low risk classification is most established for alpha-papillomaviruses, we repeated the LCR analysis using only alpha-papillomavirus sequences. Within this genus, 12 TFs were enriched in low-risk types, including members of the TEAD and SP/KLF families (Supplementary Figure S1A). TEAD factors, which regulate epithelial proliferation and differentiation programs [28,29,30], were particularly notable, as TEAD family motifs were also frequently present across beta- and gamma-papillomavirus LCRs, suggesting that TEAD-associated regulatory architectures may be a shared feature of multiple non-high-risk papillomavirus lineages (Supplementary Figure S1B). We did not find any TF motifs significantly enriched in high-risk relative to low-risk alpha-papillomavirus types.

### 3.2. Spatial Distribution and Binding Patterns of Differentially Enriched TFs Across the HPV LCR

**Figure 2:**
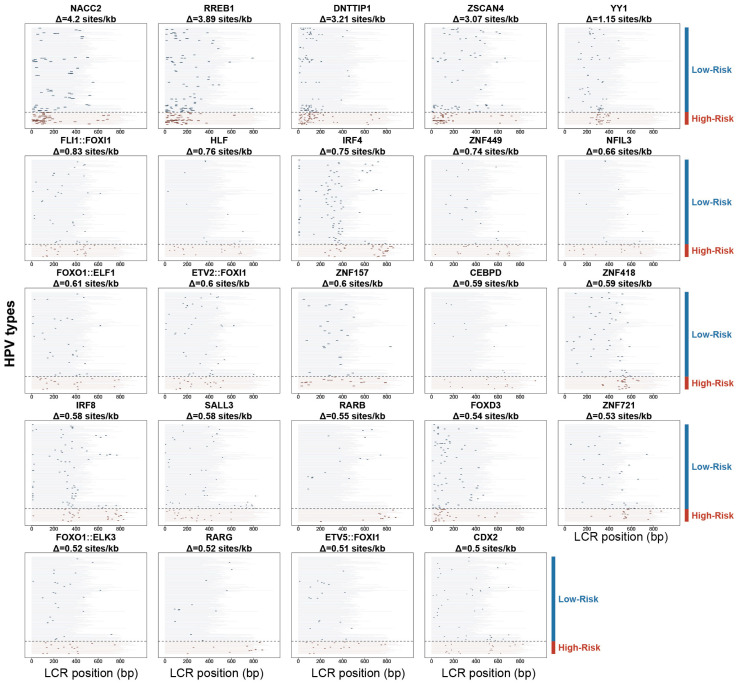
Binding positions for TFs enriched in high-risk types. Each subplot shows one TF; rows represent individual HPV types (high risk above, low risk below); marks indicate predicted binding sites along the normalized LCR. The difference in binding site density is shown below the TF names. Only TFs with a binding site density difference above 0.5 are shown.

**Figure 3:**
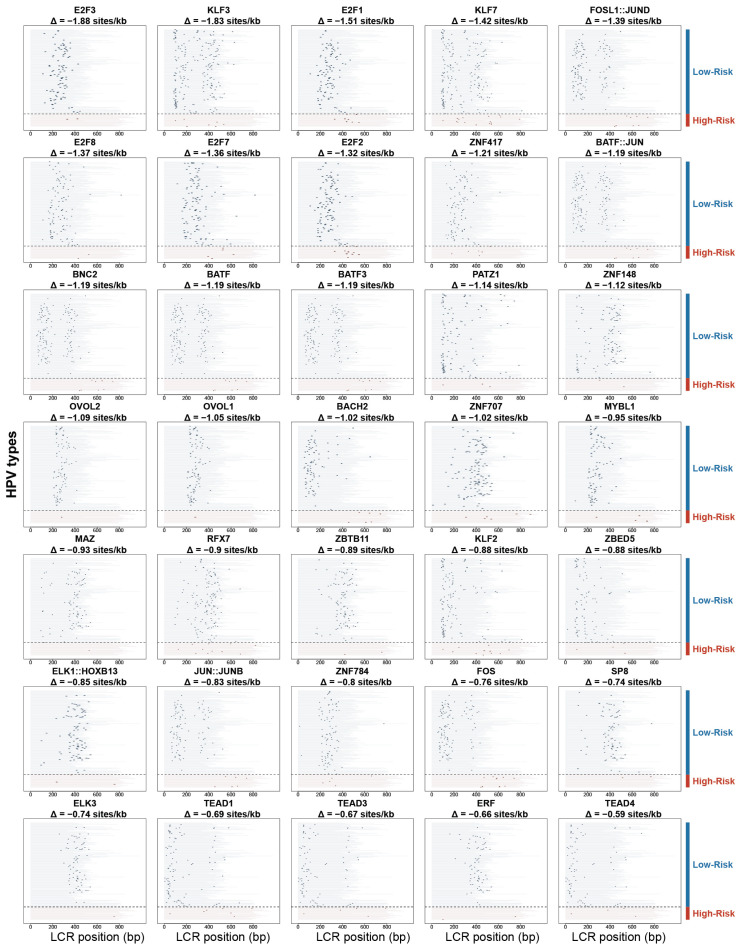
Binding positions for TFs enriched in low-risk types. Each subplot shows one TF; rows represent individual HPV types (high risk above, low risk below); marks indicate predicted binding sites along the normalized LCR. The difference in binding site density is shown below the TF names. Only TFs with a binding site density difference above 0.5 are shown.

After identifying TFs differentially enriched between high- and low-risk HPV LCRs, we next asked whether their predicted binding sites occupy conserved positions. To address this, we mapped the genomic coordinates of all predicted sites for the TFs with a mean density difference (Δ sites/kb) > 0.5 in each group and projected their positions onto a normalized LCR across 207 HPV types (Figure 2 and Figure 3). Binding sites for individual TFs were detected across many HPV types, indicating that enrichment was not driven by a small subset of genomes with unusually high site density. In high-risk types, enriched TFs were distributed throughout the LCR but showed a higher density toward the 5′ region, including factors such as NACC2, DNTTIP1, RREB1, ZSCAN4, and FOXD3 (Figure 2). While some TFs exhibited positional variability (e.g., HLF, IRF4, CDX2), most showed consistent positioning across HPV types. A comparable spatial organization was observed in low-risk LCRs (Figure 3 and Supplementary Figure S1B). Together, these results indicate that both high- and low-risk HPVs share a conserved and spatially organized LCR regulatory architecture.

### 3.3. High-Risk LCR Regulators Are Expressed in HPV-Positive Tumors

**Figure 4:**
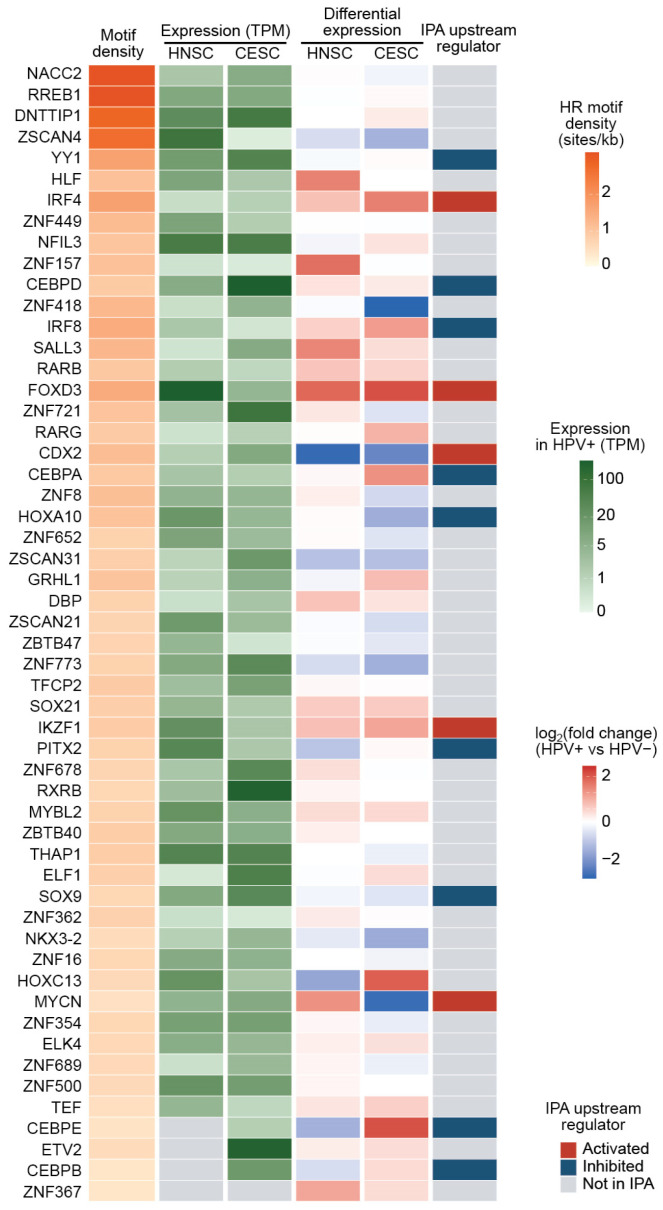
Validation summary for high-risk-enriched TFs expressed in HPV-positive TCGA-HNSC and TCGA-CESC tumors. Each row represents one of the 54 TFs enriched in high-risk HPV LCRs and expressed at TPM > 1 in ≥10% of HPV-positive patients in either HNSC or CESC. Columns show TF binding density (sites/kb) in the LCR, mean TPM in HPV-positive HNSC and CESC samples, DESeq2 log_2_(fold change) between HPV-positive and negative HNSC or CESC tumors, and IPA upstream regulator status based on differential gene expression between HPV- and HPV+ HNSC tumors. TFs are ordered by JASPAR binding enrichment.

To assess the potential functional relevance of HR-enriched TFs, we asked whether these TFs are expressed and active in HPV-positive tumors. We first cross-referenced these TFs with RNA-seq data from TCGA-HNSC (72 HPV-positive, 415 HPV-negative patients) and TCGA-CESC (167 HPV-positive, 8 HPV-negative patients), identifying 50 of 69 HR-enriched TFs as present in HPV-positive HNSC and 53 of 69 in HPV-positive CESC (TPM > 1 in ≥10% of HPV-positive tumors) (Figure 4).

We next examined whether these TFs exhibit HPV-associated expression changes that could alter LCR regulation. Among the 50 TFs in HNSC, 32 were differentially expressed between HPV-positive and HPV-negative tumors (DESeq2, padj < 0.05), including 10 with |log_2_FC| > 1 (7 upregulated and 3 downregulated), suggesting that an HPV infection may rewire host gene regulatory networks to control LCR transcriptional input. Notably, several TFs associated with transcriptional repression or epithelial differentiation, including HOXC13 [31] and the KRAB zinc-finger protein ZSCAN31, were downregulated, consistent with reduced repressive or differentiation-linked constraints at the LCR. In contrast, TFs linked to transcriptional activation or proliferative programs, such as MYCN and ZNF367 [32,33], as well as the chromatin-associated factor SALL3, were upregulated, suggesting increased availability of activator inputs that could enhance LCR activity. A parallel analysis in TCGA-CESC identified 13 of the 53 expressed high-risk-enriched TFs as differentially expressed (|log_2_FC| > 1, padj < 0.05), with 8 upregulated and 5 downregulated in HPV-positive cervical tumors (Figure 4). Of these, four TFs, including FOXD3, IKZF1, ZSCAN31, and IRF4, were differentially expressed in both HNSC and CESC, suggesting they represent HPV-associated regulatory changes shared across cancer types.

Because TF activity can change independently of expression, we next inferred upstream regulator activity using Ingenuity Pathway Analysis. Applying this approach to the HPV-positive versus HPV-negative TCGA-HNSC signature, 12 of the 50 expressed HR-enriched TFs were identified as significant upstream regulators (*p* < 0.05), with 4 predicted to have increased activity and 8 decreased activity in HPV-positive tumors. TFs predicted to have reduced activity included known or putative repressors and differentiation-associated factors such as YY1, multiple C/EBP family members (CEBPA, CEBPB, CEBPD), and SOX9, consistent with diminished inhibitory input at the LCR. In contrast, MYCN and IKZF1 showed increased expression and activity, indicating enhanced activator input from proliferative-associated TFs.

Together, these results suggest that HPV-positive tumors exhibit a shift in LCR regulatory input characterized by reduced repressive and differentiation-associated TF activity alongside increased availability and activity of transcriptional activators. This rebalanced TF landscape is consistent with a regulatory environment that favors increased or altered HPV LCR activity.

### 3.4. Evolutionary Conservation of Regulatory TFs Across HPV-16 and HPV-18 Isolates

**Figure 5:**
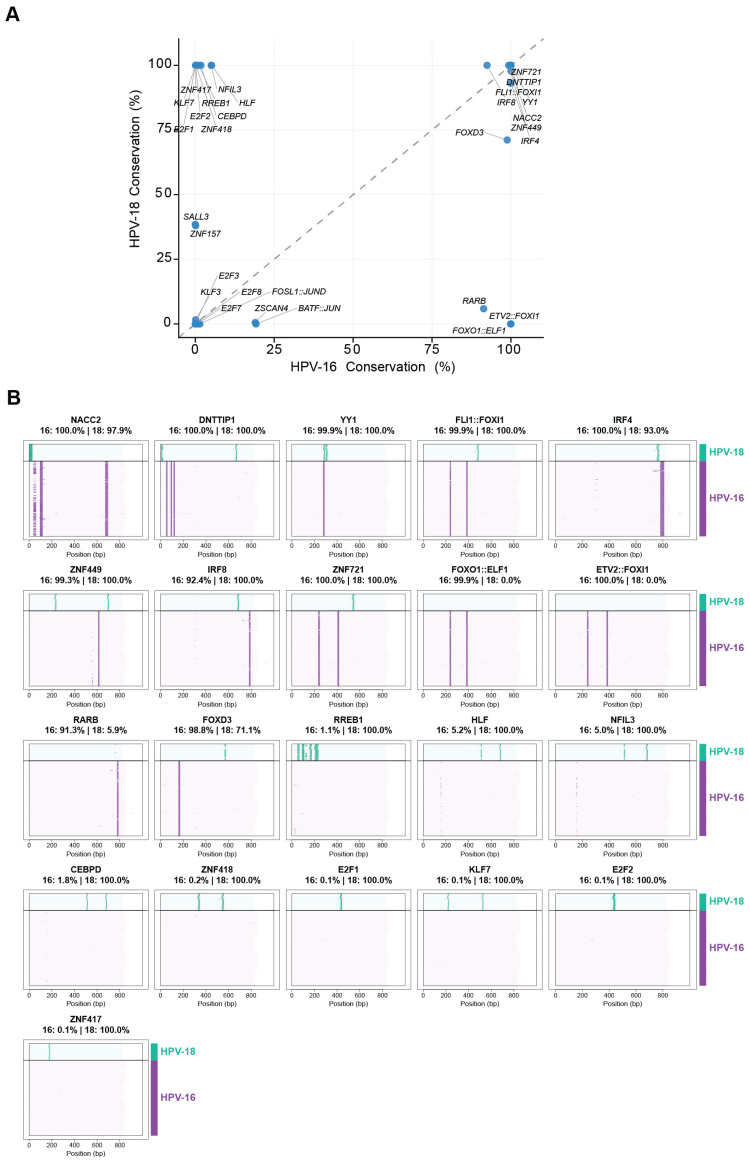
TF binding site conservation across HPV-16 and HPV-18 clinical isolates. (**A**) Scatter plot comparing conservation percentages between HPV-16 (*n* = 835 isolates, *x*-axis) and HPV-18 (*n* = 187 isolates, *y*-axis). (**B**) TF conservation grids showing binding site presence/absence across individual HPV-16 and HPV-18 isolates for TFs with greater than 90% conservation across all isolates in at least one HPV type.

To determine whether the TF binding site enrichments identified across high-risk HPV types represent functionally important features under evolutionary constraint, we performed intra-type conservation analysis by scanning 835 HPV-16 and 187 HPV-18 isolate LCRs. These isolates represent complete HPV genome sequences deposited in NCBI GenBank, collected from clinical samples across multiple countries and years. Conservation patterns differed substantially between HPV-16 and HPV-18 (Figure 5A). In HPV-16, several TFs had perfect or near-perfect conservation across isolates (99.88–100%), including IRF4, NACC2, DNTTIP1, ETV2::FOXI1, ZNF721, YY1, FOXO1::ELF1 and FLI1::FOXI1 (Figure 5A). In HPV-18, conservation was notably broader: binding sites for 15 TFs were detected in 100% of isolates, including both cross-type conserved TFs (DNTTIP1, ZNF721, YY1, FLI1::FOXI1, ZNF449, IRF8) and TFs infrequent in HPV-16 LCRs (RREB1, HLF, NFIL3, CEBPD, ZNF418, E2F1, KLF7, E2F2, ZNF417). This high conservation is expected as HPV-16 and HPV-18 isolates showed mean pairwise LCR identities of 97.49% and 98.04% respectively.

Eight TFs were conserved at ≥90% across both HPV types: DNTTIP1, YY1, FLI1::FOXI1, ZNF449, NACC2, ZNF721, IRF4 and IRF8 representing likely shared core regulatory requirements for both HPV-16 and HPV-18 (Figure 5).

Of the eight TFs conserved at ≥90% across both HPV-16 and HPV-18, seven were expressed in HPV-positive CESC tumors, and IRF4 was expressed in both CESC and HNSC, supporting their biological relevance in the context of HPV-associated cancer. FOXD3 showed a somewhat divergent pattern, with high HPV-16 conservation (98.8%) but only moderate HPV-18 conservation (71.1%). Three TFs showed high HPV-16-specific conservation with absent or minimal HPV-18 detection: ETV2::FOXI1, FOXO1::ELF1 and RARB, suggesting HPV-16-specific regulatory requirements. Conversely, nine TFs were exclusively highly conserved in HPV-18 (RREB1, HLF, NFIL3, CEBPD, ZNF418, E2F1, KLF7, E2F2, ZNF417), each with less than 6% conservation in HPV-16, pointing to a distinct set of core HPV-18 TFs (Figure 5A). These TF binding sites occupy conserved positions across isolates of the same HPV type (Figure 5B). Together, these results show that HPV-16 and HPV-18 LCRs share a common set of conserved TF binding sites, while maintaining consistent, type-specific differences that likely reflect functional divergence between HPV types.

### 3.5. TF Binding Site Enrichment in the HPV Late Promoter

**Figure 6:**
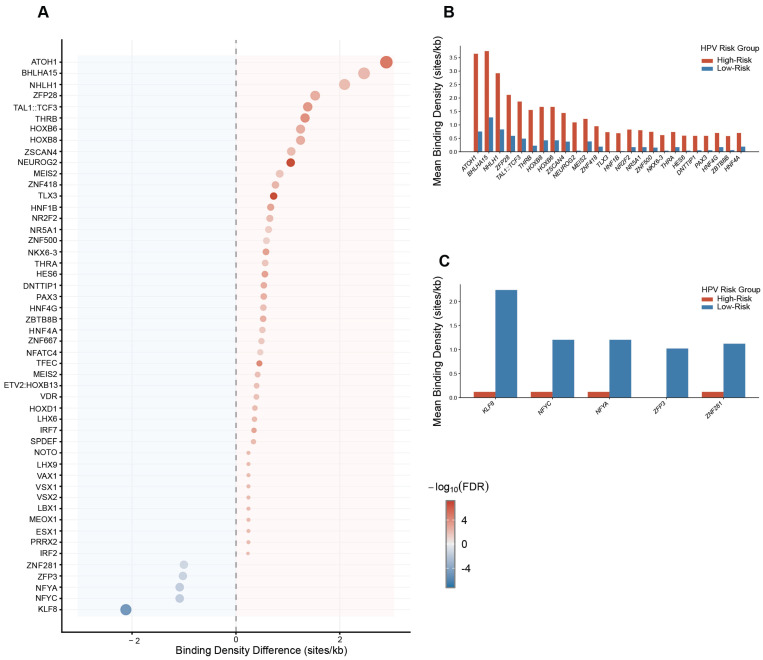
TF binding site enrichment in the HPV late promoter region. (**A**) Dot plot showing TF motif enrichment in the late promoter of high-risk (HR) versus low-risk (LR) HPV types. Dot color indicates binding density difference (HR − LR sites/kb) and dot size reflects the magnitude of the difference. Only TFs with |Difference| > 0.5 and FDR < 0.05 are shown. (**B**,**C**) Bar plots of mean binding site density (sites/kb) for HR (red) and LR (blue) HPV types for each HR-enriched (**B**) and LR-enriched (**C**) TF.

Given that the late promoter drives capsid gene expression during epithelial differentiation [3,34], we asked whether high-risk and low-risk HPV types also differ in late promoter TF binding. Using FIMO, we scanned the late promoter of 220 HPV types and identified known regulators such as YY1, CEBP, and KLF factors [35,36,37,38]. By performing differential TF motif enrichment analysis, we identified 50 TFs with significantly different binding densities between risk groups (FDR < 0.05), with 45 TFs enriched in high-risk and 5 TFs in low-risk types (Figure 6 and Supplementary Figure S2).

Consistent with the differentiation-dependent function of the HPV late promoter, many of the TFs enriched in high-risk HPV types are associated with developmental or lineage-specifying transcriptional programs, particularly bHLH and homeobox regulators, including ATOH1, BHLHA15, NHLH1, NEUROG2, HOXB6, HOXB8, TLX3, and NKX6-3 [39,40,41,42,43]. Several of these factors recognize related E-box motifs, suggesting that high-risk late promoters may preferentially recruit bHLH-associated regulatory inputs. In particular, BHLHA15 has been shown to promote cervical cancer cholesterol synthesis and tumor progression [39]. Additional enriched TFs such as HNF1B and VDR are linked to epithelial differentiation, immune signaling, or cell-state transitions [44,45]. In contrast, low-risk HPV late promoters were enriched for KLF8 and NFY complex motifs (NFYA/NFYC), factors associated with epithelial transcriptional regulation and promoter architecture [46,47]. Because the HPV late promoter is activated during keratinocyte differentiation and controls expression of late viral functions, including capsid production, these differences suggest that high-risk and low-risk HPV types may engage distinct differentiation-associated host transcriptional programs during the productive phase of the viral life cycle.

## 4. Discussion

This study provides a systematic comparison of TF binding landscapes across HPV LCRs, showing that high-risk and low-risk types are associated with distinct host regulatory repertoires. Because the LCR controls expression of the viral oncogenes E6 and E7, these differences in predicted TF recruitment suggest that variation in host regulatory inputs may contribute to the divergent transcriptional programs and pathogenic outcomes of HPV infection.

The distinct TF binding landscapes observed in the LCRs of high-risk types may reflect adaptations to specific epithelial and transcriptional environments associated with persistent infection, with oncogenic transformation emerging as a downstream consequence of these regulatory programs. Several enriched TFs have established roles in oncogenic signaling or in HPV-associated cancers, pointing to a regulatory context that may favor sustained proliferation and altered cell state. YY1 provides a clear example of how such factors may act in a context-dependent manner, functioning as an HPV-18 LCR repressor in most cell types but switching to an activator in cervical carcinoma cells through interaction with C/EBPβ [13,26]. Several TFs enriched in high-risk LCRs have established roles in oncogenic signaling or cancer-associated regulatory programs. NACC2 is a transcriptional repressor that recruits the NuRD/HDAC complex to silence target gene promoters; it stabilizes p53 by repressing HDM2 and acts as a tumor suppressor whose loss promotes oncogenic HDM2 activity [48,49]. RREB1 is overexpressed across multiple cancer types and promotes epithelial-to-mesenchymal transition and tumor invasion by cooperating with TGF-β–activated SMADs downstream of MAPK signaling [50,51]. DNTTIP1, a subunit of the HDAC1-containing MiDAC complex, has established oncogenic roles in oral squamous cell carcinoma and nasopharyngeal carcinoma, where it recruits HDAC1 to silence DUSP2 and promote ERK-driven metastasis [52,53,54]. ELK1 and ELK4 are ETS-family TFs that have been directly linked to HPV-positive cervical cancer through interactions with viral oncoproteins [55,56]. ZSCAN4, which promotes telomere elongation and histone acetylation at pluripotency gene promoters, has been shown to expand the cancer stem cell population in HNSCC [57,58]. In contrast, the enrichment of E2F and AP-1 family members in low-risk LCRs is consistent with low-risk HPV replication depending on the host epithelial differentiation pathway rather than persistent oncogenic transformation [7,59]. E2F1, E2F2 and E2F3 are canonical-activating E2F family members that promote G1/S transition [60], whereas E2F7 and E2F8 are transcriptional repressors that suppress E2F-target genes [61]. The E2F family is among the most widely dysregulated transcriptional regulators in human cancer [62]. Their enrichment in low-risk rather than high-risk LCRs suggests that E2F binding may have a role in supporting viral replication in differentiating keratinocytes, rather than driving oncogenic transformation [63]. Within alpha-papillomaviruses specifically, 12 TFs were enriched in low-risk types, including TEAD and KLF family members, while no TFs reached significance in high-risk types. TEAD family members are the primary transcriptional effectors of the Hippo/YAP pathway [30], which controls proliferation and differentiation decisions in stratified keratinocytes [29]. Their enrichment in low-risk alpha-papillomavirus LCRs may reflect adaptation to the normal keratinocyte transcriptional environment, consistent with low-risk HPV’s dependence on intact host epithelial biology for productive infection. Among the 7 TFs found with CHIP-seq evidence for LCR peak enrichment, YY1 and C/EBPβ were shown to bind the HPV-18 upstream regulatory region [13,26,64]. The remaining five TFs have not been shown to occupy the HPV-18 LCR. DNTTIP1 was recently shown to directly contact the TATA-box region of the HIV-1 core promoter [65], suggesting a possible mechanism for engagement of the TATA-dependent HPV-18 promoter. ELK1 has been shown to physically interact with HPV-18 E7 in cervical cancer cells, synergistically driving proliferative gene expression, including EGR1 and c-FOS [55], while ELK4 is overexpressed in HPV-positive cervical tumors and promotes cell cycle progression and stem cell-like properties through FBXO22-mediated PTEN degradation [56]. MYBL2 is a transcriptional activator that, in complex with MuvB, drives expression of late cell cycle genes during S phase [66]. HPV-16 E7 deregulates MYBL2 through two mechanisms: disruption of p107/E2F-mediated repression at the MYBL2 promoter [67] and direct engagement of the MYBL2-MuvB-FOXM1 complex to amplify mitotic gene expression [66].

The conservation analysis further supports the functional importance of the regulatory differences identified here. A subset of TF binding sites enriched in high-risk LCRs is also highly conserved across HPV-16 and HPV-18 isolates, despite overall sequence variability within the LCR. This pattern argues against neutral sequence drift and instead points to selective constraint on specific regulatory elements. The observation that the same TF binding sites are maintained across two genetically distinct high-risk types suggests the existence of a shared regulatory core that may be required for efficient control of viral transcription. At the same time, the presence of type-specific TFs conserved across isolates indicates that this core is complemented by lineage-specific regulatory features, potentially contributing to differences in viral behavior even among high-risk types.

Independent lines of evidence support the biological relevance of the predicted TF binding differences. First, more than 70% of high-risk-enriched TFs are expressed in HPV-positive tumors, indicating that they are present in the cellular context where LCR regulation occurs, many of which are differentially expressed in HPV+ tumors. Second, ChIP-seq data demonstrate physical occupancy of several of these factors at the HPV-18 LCR, linking motif predictions to in vivo binding. Finally, differentially expressed genes in HPV+ tumors are predicted to be targets of 12 TFs enriched in high-risk types. While expression does not guarantee binding and ChIP-seq coverage remains limited, the convergence of motif enrichment, conservation, expression, and occupancy supports the idea that these TFs represent bona fide components of the HPV LCR regulatory network.

Several limitations should be considered when interpreting these results. TF binding site predictions based on position weight matrices do not necessarily indicate in vivo TF binding, and do not capture cooperative interactions, chromatin accessibility, or cell type-specific regulatory context [68]. Moreover, the available ChIP-seq data are restricted to HeLa-derived cells and may not fully reflect TF occupancy in primary infection settings. Furthermore, while ChIP-seq confirms physical TF-DNA interaction, it does not demonstrate that bound TFs actively regulate viral gene expression. Demonstrating this directly would require loss-of-function experiments, such as TF siRNA knockdown or CRISPR-based editing of TF binding sites, to assess the effect on viral transcription. Future work will be required to determine which of the identified TFs directly regulate LCR activity and how their binding influences viral gene expression across types and isolates. Experimental approaches such as reporter assays, targeted perturbation of binding sites, and profiling of TF occupancy in primary HPV-infected cells will be important to establish causal relationships.

Additionally, because oncogenicity itself is unlikely to represent the primary evolutionary pressure acting on papillomaviruses, future unsupervised analyses of TF binding architectures across HPV types may reveal regulatory groupings associated with tissue tropism, epithelial differentiation programs, persistence strategies, or viral phylogeny beyond what is captured by the high-risk/low-risk classification used here. Such approaches may help distinguish TF networks associated with epithelial niche adaptation from those secondarily associated with oncogenic potential.

In summary, this study identifies widespread and systematic differences in TF binding potential between high-risk and low-risk HPV LCRs and highlights a set of conserved regulatory elements associated with oncogenic types. These findings provide a framework for understanding how variation in host TF recruitment may shape viral transcriptional programs and contribute to HPV pathogenicity, and they define a set of candidate regulators for future functional investigation.

## Data Availability

Data are contained within the article and Supplementary Materials. Code used to perform data analysis is provided as Supplementary file: Supplementary_code.zip.

## Supplementary Materials

The following supporting information can be downloaded at: https://www.mdpi.com/article/10.3390/v18060646/s1, Figure S1: TF binding site enrichment in the LCR of alpha-papillomaviruses. (A) Dot plot showing TF motif enrichment in the long control region (LCR) of high-risk (HR) versus low-risk (LR) alpha-papillomavirus types (26 HR vs. 31 LR). Dot color indicates binding density difference (HR−LR sites/kb) and dot size reflects the magnitude of the difference. (0 HR-enriched, 12 LR-enriched). (B) Binding site distributions of low-risk-enriched TFs across alpha-papillomavirus LCRs. Positions of TF binding sites within the long control region (LCR) for all LR-enriched TFs (|Difference| > 0.5 sites/kb, FDR < 0.05), shown across alpha-papillomavirus HPV types. Each row is one HPV type; Figure S2: TF binding site distributions in the late promoter. (A,B) Binding site distributions of high- (A) and low- (B) risk-enriched TFs across HPV types. (Difference > 0.5 sites/kb, FDR < 0.05), shown across all HPV types. Each row is one HPV type; Table S1: List of HPV types, accession numbers, and risk used in this manuscript; Table S2: List of accession numbers for HPV-16 and HPV-18 isolates used in this manuscript; Table S3: Motif analysis results presented in this manuscript.
