# Supplementary figures and images for: Comparative Analysis of Transcription Factor Binding Sites in the Long Control Region Across Human Papillomavirus Types
Source: Viruses. 2026 Jun 4;18(6):646. doi: 10.3390/v18060646 (PMC13308338; doi:10.3390/v18060646)

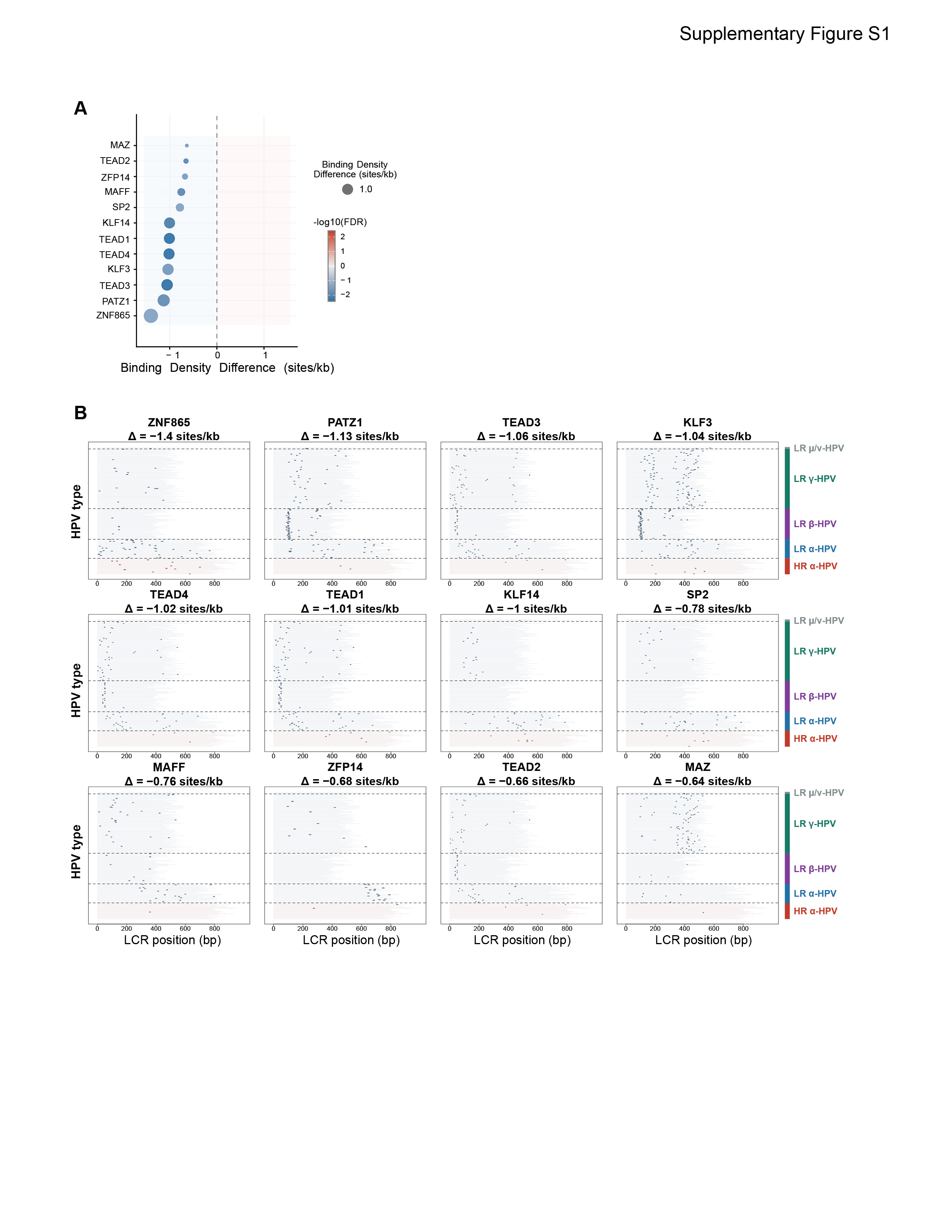

Supplement: Supplementary file 1 [file viruses-18-00646-s001.zip › Figure S1.jpg]

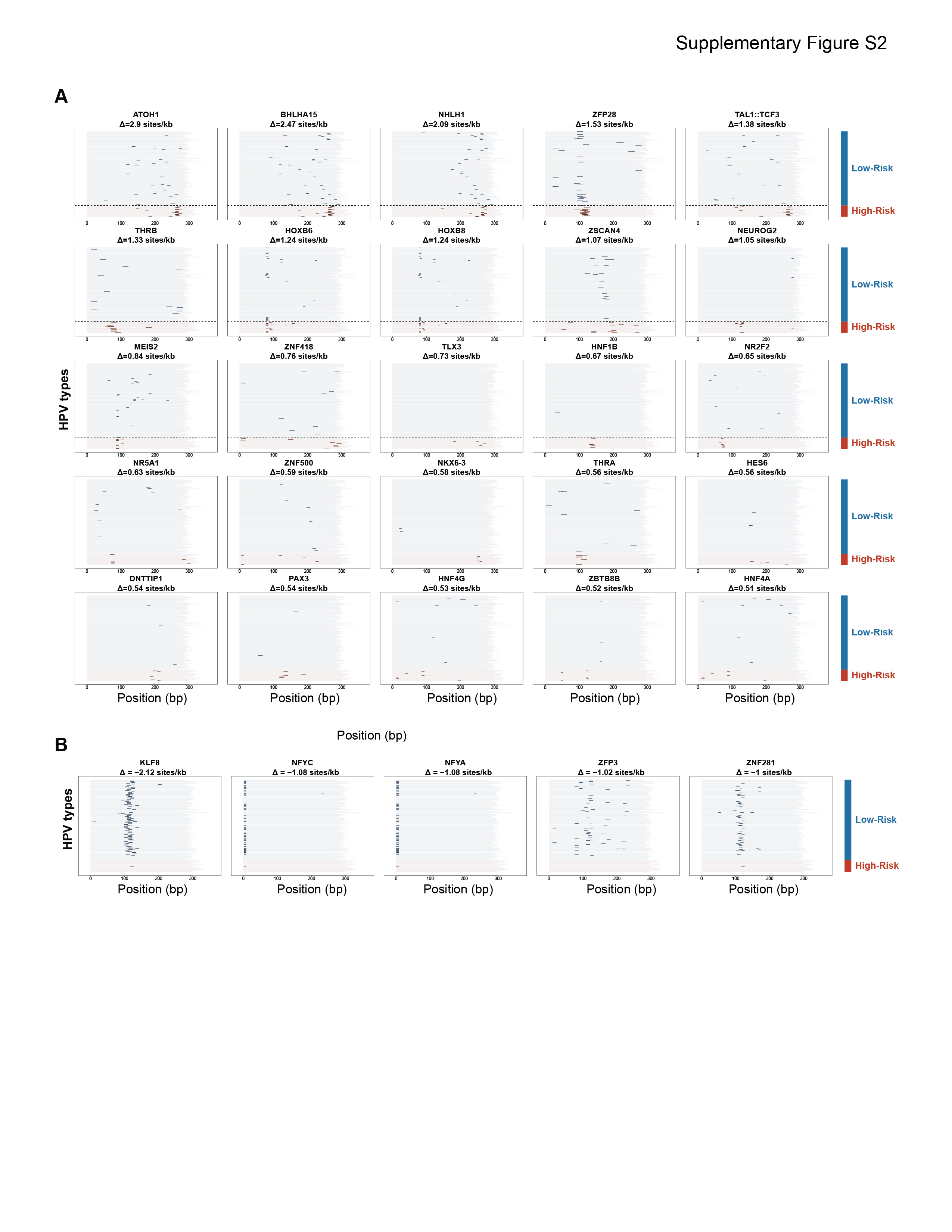

Supplement: Supplementary file 1 [file viruses-18-00646-s001.zip › Figure S2.jpg]
